# Providing a Forensic Expert Opinion on the “Degree of Force”: Evidentiary Considerations

**DOI:** 10.3390/biology10121336

**Published:** 2021-12-16

**Authors:** Hans H. de Boer, Charles E. H. Berger, Soren Blau

**Affiliations:** 1 Victorian Institute of Forensic Medicine/Department of Forensic Medicine, Monash University, Southbank, VIC 3006, Australia; soren.blau@vifm.org; 2 Institute of Criminal Law and Criminology, Leiden University, P.O. Box 9500, 2300 RA Leiden, The Netherlands; C.Berger@nfi.nl; 3 Netherlands Forensic Institute, Ministry of Justice and Security, Government of The Netherlands, 2511 DP The Hague, The Netherlands

**Keywords:** degree of force, skeletal trauma, forensic pathology, forensic anthropology, review, evidence, opinion, likelihood ratio, Bayes’ theorem

## Abstract

**Simple Summary:**

When giving evidence in court, forensic pathologists and anthropologists are often asked for their opinion on the amount, or degree of force required to cause a specific injury. Such ‘degree of force’ questions are considered difficult, if not impossible to answer due to many theoretical and practical issues. This paper explores these issues and provides a possible solution. First, the logical underpinnings of the question on the ‘degree of force’ are explored. Then the experimental research on ‘degree of force’ is reviewed and the limitations with applying this research to everyday forensic casework are discussed. In the second part of the paper, it is argued that these limitations do not, however, mean that a forensic pathologist or anthropologist cannot add anything of value to the discussion. The application of Bayes’ theorem helps to circumvent many of the problems. The final part of the paper is dedicated to a detailed discussion of how it can be applied to the issue of ‘degree of force’.

**Abstract:**

Forensic pathologists and anthropologists are often asked in court for an opinion about the degree of force required to cause a specific injury. This paper examines and discusses the concept of ‘degree of force’ and why it is considered a pertinent issue in legal proceedings. This discussion identifies the implicit assumptions that often underpin questions about the ‘degree of force’. The current knowledge base for opinions on the degree of force is then provided by means of a literature review. A critical appraisal of this literature shows that much of the results from experimental research is of limited value in routine casework. An alternative approach to addressing the issue is provided through a discussion of the application of Bayes’ theorem, also called the likelihood ratio framework. It is argued that the use of this framework makes it possible for an expert to provide relevant and specific evidence, whilst maintaining the boundaries of their field of expertise.

## 1. Introduction

*“… force alone is woefully inadequate and often (particularly in a legal environment) misleading in describing an impact”.*

[1], p. 283

The concept that skeletal trauma occurs when a force exceeds the strength or maximum threshold of bone elasticity is well established [2,3]. In forensic pathology and anthropology, descriptions of the application of a force to the body are typically divided into three groups of causation: blunt force, sharp force and high or low energy ballistic force. While the potential results of these forces on the human body have been well documented in the biomechanical [3,4,5] and forensic medical literature [1,6,7], correlating the amount of force applied to the body to a specific injury or fracture outcome has proven more difficult. Nonetheless, when providing expert opinion in court about skeletal injury, forensic pathologists and anthropologists are often asked their opinion about the cause and specifically, the amount or the ‘degree of force’ that was required.

The relationship between injury morphology and applied force is complex, and opinions on the ‘degree of force’ are therefore fraught with difficulties. This paper provides an overview of the concept of ‘degree of force’ in forensic pathology and anthropology and in doing so, provides an aid for practitioners when giving evidence on this issue. While the paper focuses on skeletal injuries, much of the discussion also applies to the same issue when interpreting soft tissue injuries.

## 2. Why Is the ‘Degree of Force’ Considered to Be Important?

In order to understand why the issue of ‘degree of force’ appears to be so pertinent in criminal cases it is necessary to reflect briefly on the purpose of a criminal court proceeding, and the role of the expert witness. Although differences between jurisdictions exist, a criminal court proceeding typically aims to determine whether enough evidence is available to convict the defendant for the alleged crime. This process is traditionally dialectic, with prosecution and defence both trying to convince the trier-of-fact of their respective positions, usually by presenting evidence. The opinion of the expert witness, like other evidence, can assist the trier-of-fact in weighing the competing positions of prosecution and defence.

Within this context, opinions on the ‘degree of force’ have been considered useful to help the trier-of-fact to reconstruct the events that led up to and resulted in death. In other words, such opinions are intended to help the trier-of-fact choose between various scenarios. The high frequency with which forensic pathologists [8,9,10,11]; forensic anthropologists, and forensic physicists [12] are confronted with the question suggests that such an opinion is considered particularly helpful by the court. This perceived value of opinions on the ‘degree of force’ appears to be based on three assumptions, namely that proportional relationships exist between:

1. The intent of an offender/assailant, and the amount of force they use;
2. The amount of force that an offender/assailant uses, and the amount of force that is actually transferred to the body of the decedent;
3. The amount of force that is applied on the body of the decedent, and the severity of injury.

If all three assumptions are valid, the conjecture is that knowing the ‘degree of force’ may help to differentiate between intentional or accidental injuries, and therefore, help to conclude if a crime was actually committed. Furthermore, since the seriousness of a crime ordinarily influences sentencing decisions [13], intent is an important aspect of culpability in many jurisdictions. An expert opinion that can inform on intent can therefore have an impact on sentencing [14].

## 3. Forensic Expert Responses to the Question of ‘Degree of Force’

Questions relating to the ‘degree of force’ may be asked in various forms. Typically, however, the expert is asked the question in a simplistic form: “what degree of force is required to cause this skeletal injury?” The expert is subsequently expected to provide an estimate of that amount of force, based on the combination of observations, knowledge, and experience.

Anecdotal information, largely obtained from discussions amongst forensic experts, indicates there is variation in their responses. The general consensus is that a specific answer (i.e., including a number expressing the amount of force) cannot be provided. As a result, experts may provide a response along the lines of “I am unable to comment” or “I can comment, but without a degree of precision”. Other types of responses include “the force was sufficient to result in a skeletal injury”; or “clearly there has been enough force to fracture a bone”. Since such comments only reiterate the facts that are already known, it may be argued that these opinions are as uninformative as “no comment”.

To simplify the issue, some experts choose to use a qualitative three- or four-point scale to describe the amount of applied force. This approach has also been described in the literature, with verbal descriptions such as “mild”, “moderate” and “severe” force used by Nolan et al. [15]; and “mild”, “moderate”, “considerable” and “severe” force by Gilchrist et al. [16] and Sharkey et al. [17]. A definition of what these specific categories mean, or how the expert should choose between them, however, remains largely undiscussed. In a study pertaining to stab injuries, Gilchrist and colleagues [16] stated that a mild level of force would “typically” be associated with penetration of skin and soft tissue, moderate force with injuries that penetrate cartilage or rib bone, and severe force with injuries that penetrate dense bone and cause visible damage to the knife’s blade. But these definitions are not generally accepted, and the limitations of using these vague and relative terms have been previously noted [8,15].

Overall, there is no consensus on how an expert should answer a question on the ‘degree of force’. This lack of consensus has served as a justification for research which has sought to quantify the degree of force in various types of injuries.

## 4. Evidence for the Relationship between Degree of Force and Injury Outcome

A range of experimental studies have been undertaken to investigate and correlate the relationship between degree of force and injury outcome. This research ordinarily focuses on the method of injury, rather than on the type of tissue injured. For instance, research has included the investigation of degree of force and sharp force trauma involving knives [8,16,18], as well as stabbing involving other implements such as screwdrivers [19]. Such research has used pork skin [15,18] as well as synthetic materials such as foam [20,21], silicone rubbers [22,23] and modelling clay [24] as substitutes for human skin. Research investigating the relationship between degree of force and blunt force trauma has also been undertaken. This research has mostly focused on understanding the force required to cause head injuries, including brain injuries [25] and skull fractures [26]. As experimental models, researchers have used human skulls [27] as well as those of pigs [17,26] and monkeys [28], in addition to computer simulations [29].

Despite the use of these various experimental models, different anatomical parts of the body, and different types and amounts of force, the results of these experimental studies are difficult to apply to forensic casework. This shortcoming becomes more apparent when reconsidering the previously mentioned three assumptions that underpin the alleged validity of the ‘degree of force’ question.

Experimental research has predominantly focused on the third assumption: the relationship between the force applied to the body, and the severity of injury. Consequently, such research only addresses one part of the issue at hand. Further, the highly controlled settings typical of experiments do not (and cannot) take into account the many intrinsic and extrinsic variables that influence the relationship between applied force and injury outcome. Intrinsic variables include the sex and age of the deceased, and the specific anatomical region impacted (e.g., head vs. chest). The anatomical region, and therefore the skeletal element, is also important to consider, as different bones differ in their density, flexibility, and design (e.g., the area of impact may be buttressed by other anatomical structures) [27]. In addition to the health status which affects bone plasticity [30], individual variation in bone morphology must also be considered (e.g., skull thickness [31,32,33]). Overall, while the results of experimental research may be interesting as a means of demonstrating the biomechanical properties of human (and non-human) tissue, they are not directly transferable to forensic casework.

Published research focused much less on the first and second assumptions that underpin the ‘degree of force’ question, that is, the relationship between ‘intent’ and ‘force used’, and between ‘force used’ and ‘force transferred’. The difference between ‘force used’ and ‘force transferred’ is an often-overlooked issue in experimental settings but is, nonetheless, relevant in forensic casework. In many instances the relationship between these two forces is not proportional. Consider, for example, a situation in which a perpetrator exerts what may be described as a ‘relatively minimal’ force (e.g., a gentle, yet intentional push) which nonetheless results in what is described as ‘severe trauma’ (e.g., multiple comminuted fractures due to a fall from height). In other settings the relationship between ‘force used’ and ‘force transferred’ may be proportional, but there is no way of knowing the extent to which one is influenced by the other. For example, in the case where a perpetrator uses an implement that modifies the force that is used (e.g., a baseball bat, a hammer, or a knife). Extrinsic variables such as the effect of the size, shape, elasticity, and mass of the impacting implement [17,27] are important in this regard. The directionality of the impact is also of interest [12] as well as its speed (because bone is viscoelastic, that is, responds differently depending on the speed at which a load is applied). The direction-ality and speed of impact relate directly to the relative position of perpetrator and victim and the dynamics of the event. When all these variables are considered, it becomes appar-ent that the same amount of force used by a perpetrator can, depending on the circumstances, result in different amounts of force being transferred (applied) to the body of the victim.

The assumed relationship between the intent of the perpetrator and the force that is used is also rarely considered in empirical research. Although it is intuitively true that the intention to inflict grievous bodily harm results in a large amount of force being used, it is not necessarily so that unintentional behaviour results in less force. Consider, for example, scenarios of self-defence, in which forcefully fending off an attack can cause serious harm to the attacker. Moreover, one study showed that when volunteers were asked to use ‘mild’, ‘moderate’ or ‘severe’ force, the resultant amounts of (stabbing) force were too similar to reliably infer the ‘intent’ of the volunteer [15]. The sex and age of the perpetrator have been noted as important variables to consider in this [12,15]. However, these are only two of a multitude of interacting variables that may be of relevance.

Overall, while the findings from experimental research can perhaps support claims about the potential effects of force on the human body, the data seem of limited use to provide informative opinions on the ‘degree of force’. It may be argued that, in fact, the results from experimental research reinforce the idea that the ‘degree of force’ is an issue associated with great complexity and uncertainty, while its relevance is very limited.

## 5. Taking a Different Approach: Applying Bayes’ Theorem

Given the complexity and uncertainty associated with providing an opinion on the ‘degree of force’, an alternative approach is to use probability, described as “a tool to handle uncertainty” [34]. The difficulties surrounding the issue can perhaps be addressed by applying the laws of logic and probability, using Bayes’ theorem. This theorem describes the logical underpinnings of the process by which probabilities are updated based on observations [35]. Many textbooks and journal articles provide introductions to Bayes’ theorem, and explain why its use is the logically correct way to interpret and present forensic evidence [34,36,37,38]. Bayes’ theorem has been applied in a range of forensic disciplines including pathology [39,40], anthropology [41,42], entomology [43], biometrics [44], and biomechanics [45], and to address different questions such as time since death [46], manner of death [47], and identification [48,49], including disaster victim identification [50,51,52] and missing persons investigations [53]. To date, however, Bayes’ theorem has not yet been applied to address the issues associated with opinions on the ‘degree of force’.

Bayes’ theorem, which in forensic science is also referred to as ‘the likelihood ratio framework’, is best explained by the equation in odds form:

$$\frac{P\left(H1\right)}{P\left(H2\right)}\times \frac{P(E|H1)}{P(E|H2)}=\frac{P(H1|E)}{P(H2|E)}$$

With:

P(Hx) = prior probability of proposition x

P(*E*|Hx) = probability of the evidence *E*, given proposition x

P(Hx|*E*) = posterior probability of proposition x, i.e., given the evidence *E*

This can also be written as:

prior odds × likelihood ratio = posterior odds

It should, however, be kept in mind that this equation only shows the logical relationship between the probabilities of observations and propositions. The theorem therefore remains valid in the absence of numerical data.

### 5.1. Prior Odds

The prior odds are given by the ratio of the probability of proposition H1 and that of H2, without considering the expert’s observations, that is, the evidence (*E*). Because the prior odds are based on all information outside the expert’s evidence, assessing the prior odds would take the expert outside their area of expertise.

### 5.2. Likelihood Ratio

To provide an opinion while staying within their area of expertise, the expert needs to focus on the likelihood ratio (LR) only. The LR is the ratio of two probabilities: the probability of their observations (*E*) given one proposition is true, and the probability of the same observations given an alternative (mutually exclusive) proposition is true. Assessing these two probabilities relies directly on the experience and expertise of the expert. This process does not necessarily imply using statistics and calculations: the same logic applies with or without the use of numerical data.

### 5.3. The Posterior Odds

The posterior odds take all the evidence into account: they equal the prior odds multiplied by the LR. Since the posterior odds require the prior odds, the posterior odds are also outside the forensic pathologist or anthropologist’s area of expertise.

## 6. A Hypothetical Case Example

The utility of the application of this framework to the issue of ‘degree of force’ can be illustrated by the following hypothetical case. The partially skeletonized remains of an adult male were located at the bottom of a mine shaft. The individual’s skull was fragmented. The remains were examined by a forensic pathologist and a forensic anthropologist. Reconstruction of the skull fragments revealed two concentric, patterned impact fractures: one in the left fronto-temporal region, and the other in the left temporo-parietal region. There was also a linear defect on the right posterior aspect of the occipital bone. In their joint report, the forensic pathologist and anthropologist concluded that these observations indicated multiple impacts, and that the cranial trauma was a reasonable cause of death. Eventually, a person was arrested in relation to the matter and the case went to trial. In court, the experts were asked their opinion on the ‘degree of force’ required to produce this fragmentation and patterned injury.

While the experts can try to answer this question, as previously discussed, many limitations preclude the provision of a robust opinion. Using vague terms such as ‘mild’, ‘moderate’, ‘severe’ and ‘extreme’ to describe force does not overcome these limitations. These restrictions do not, however, mean the expert cannot add anything of value to the discussion.

## 7. The Need for Propositions

When applying Bayes’ theorem, the expert’s opinion is used as evidence to help give weight to one of two propositions, most often the positions of the prosecution and defence. For instance, in the hypothetical case outlined above, the prosecution may allege that the decedent was beaten to death with a shovel and then dumped in the mine shaft. In contrast, the defence may propose that the skull fractures were the result of a fall following a verbal altercation between the decedent and the defendant. As discussed previously, information about the ‘degree of force’ is just an intermediate step in addressing the larger issue: which of the two propositions is correct. If the court is focused on one specific injury and only enquires about the ‘degree of force’ required, these propositions are not made explicit to the expert. Consequently, the full meaning of the pathological/anthropological findings cannot be borne out.

Only when provided with propositions, can the expert provide the most relevant evidence. For instance, in the hypothetical case, the expert could clarify that the propositions provided by prosecution and defence both imply that substantial force was applied to the skull, and therefore, an opinion on the ‘degree of force’ is of no use to distinguish between the two propositions. Further, by focusing on ‘degree of force’, other observations made by the expert remain undisclosed. In the hypothetical case example such information includes the findings that the victim had a minimum number of three impacts, both sides of the skull were impacted, and that there were two patterned impression fractures and one linear fracture. These details are all potentially useful to the court proceedings, especially when the expert is provided with some case circumstances.

## 8. How Does the Expert Assess Evidential Strength (An LR)?

Instead of requesting an opinion on the ‘degree of force’, a more appropriate question for the expert may be: “to what extent do your observations support scenario A (that the decedent was assaulted with a shovel and dumped in the mineshaft) vs. scenario B (that the decedent fell into the mineshaft)?” When confronted with these propositions, the expert can apply Bayes’ theorem, and therefore provide the evidential strength of their observations (an LR).

But how are experts supposed to assess an LR? Where do they get ‘the numbers’ from? It is important to remember that the use of probability does not imply statistics and calculations [34], and that a lack of data does not preclude the application of logic. LRs can be used qualitatively. However, the LR framework cannot mitigate gaps in scientific knowledge. If the expert thinks there is insufficient scientific knowledge to provide an opinion, it is their professional obligation to say so. In that situation the expert’s opinion represents an LR of 1, which simply means that in the expert’s opinion, their observations do not assist in distinguishing between the two propositions.

In the hypothetical case the observations of the two concentric, left-sided patterned impact fractures in the fronto-temporal and temporo-parietal regions, and the right-sided linear defect in the occipital region are the relevant evidence (*E*). The first question is, therefore, to what extent does the expert expect (or is surprised by) these observations if scenario A (H1) is true? How probable is the presence of a linear fracture when hit with a shovel? And would such an impact result in multiple concentric, patterned impact fractures? Moving to scenario B (H2), what is the probability of the observations if the deceased just fell in the shaft without being beaten? Answers to these questions rely on the expert’s observations. Ideally, however, they would also be informed by some (preferably undisputed) information on the case circumstances. In this case this information would include details about the structure (walls and bottom), height, and width of the mine shaft. To obtain an LR the expert finally needs to relate the expectation for the observations under both propositions to one another, because it is their ratio that determines the evidential strength. It is important to remember that having a low expectation for the observations under one proposition does not imply support for the other proposition, since the observations could be even more improbable under the alternative proposition.

Suppose that in the hypothetical case the mine shaft was dug into soil, did not contain any rocks, and was six meters deep. In these circumstances there is a much higher expectation for the three fractures under scenario A (the decedent was assaulted with a shovel and dumped in the mineshaft) than under scenario B (the deceased just fell in the shaft without being beaten). Suppose the probability of the observations is assessed to be higher by a factor of hundreds for scenario A versus scenario B. If the opinion scale as defined in the ‘Guideline for Evaluative Reporting’ by the European Network of Forensic Science Institutes (ENFSI) [54] is used, LRs in the range between 100 and 1000 are represented as ‘moderately strong support’. With reference to that scale, the expert would report that the observations offer ‘moderately strong support’ for scenario A over scenario B. The ‘moderately strong support’ is the qualitative LR in this example. This LR does not imply that scenario A is the most probable scenario, as other evidence (prior odds) could point to scenario B. It does, however, mean that this expert opinion offers moderately strong support for the case of the prosecution. How to best communicate (verbal) LRs is discussed in more detail in [55].

Note how the propositions enable the expert to use all their observations to answer the question, instead of focusing on one (often out of context) single element (i.e., the degree of force). This approach increases the amount of information that can be used for the opinion. Instead of being constrained to the limited empirical evidence for the relationship between force and injury morphology, the expert can now use other sources of information as well. For instance, the expert can refer to published literature which provides an evidence base for the types of skull fractures associated with different categories of trauma (e.g., [27,56]), or fracture patterns, i.e., the number, location and morphology of skull fractures in falls [57] vs. assaults [58].

Making the question explicit in the form of propositions allows the expert to provide an LR. It furthermore clarifies the issues most relevant to the court and therefore allows the expert to maximize the relevance of their evidence. Moreover, as previously discussed in various other publications dedicated to the application of Bayes’ theorem in forensic science, it helps to maintain the separate roles of the trier-of-fact and the expert, and helps to interpret evidence in a logically correct way. Thus, when asked the right question, the expert can appropriately draw on their expertise and therefore, inform the court in the most meaningful way.

## 9. Conclusions

Questions relating to the ‘degree of force’ often implicitly assume that such an opinion assists the court in establishing whether an injury was caused accidentally or intentionally. As demonstrated in this paper, this assumption is flawed, since theoretical and practical limitations preclude a connection between the ‘degree of force’ and intent. Similar to forensic biomechanical injury assessment, providing an opinion about the ‘degree of force’ does not occur in a vacuum [45], that is, all lines of evidence must be considered. The use of Bayes’ theorem helps to accomplish this, and therefore enables the expert to maximize the full potential of their evidence.

## Data Availability

Not applicable.
